# Electrospinning Live Cells Using Gelatin and Pullulan

**DOI:** 10.3390/bioengineering7010021

**Published:** 2020-02-22

**Authors:** Nasim Nosoudi, Anson Jacob Oommen, Savannah Stultz, Micah Jordan, Seba Aldabel, Chandra Hohne, James Mosser, Bailey Archacki, Alliah Turner, Paul Turner

**Affiliations:** 1 Department of biomedical engineering, College of Engineering and Computer Sciences (CECS), Marshall University, Weisberg Family Applied Engineering Complex, Huntington, WV 25755, USA; 2 Wright State University, Biomedical, Industrial and Human Factors Engineering, 228 Russ Engineering, 3640 Colonel Glenn Hwy, Dayton, OH 45435, USA

**Keywords:** electrospinning, live-cell electrospinning, tissue engineering, cell seeding, high voltage, viability

## Abstract

Electrospinning is a scaffold production method that utilizes electric force to draw a polymer solution into nanometer-sized fibers. By optimizing the polymer and electrospinning parameters, a scaffold is created with the desired thickness, alignment, and pore size. Traditionally, cells and biological constitutes are implanted into the matrix of the three-dimensional scaffold following electrospinning. Our design simultaneously introduces cells into the scaffold during the electrospinning process at 8 kV. In this study, we achieved 90% viability of adipose tissue-derived stem cells through electrospinning.

## 1. Introduction

Tissue engineering aims to produce synthetic tissues that maintain, restore, or improve native tissue functions [1]. Engineers utilize the formation of both acellular scaffolds and scaffolds that are seeded with cells to accomplish these objectives. Acellular scaffolds are typically used to define a space for new tissues to develop [2]. These scaffolds serve as an extracellular matrix to promote cell adhesion and growth in vivo. Scaffolds with seeded cells have a greater significance because they closely mimic human tissues. It is essential for cell adhesion and migration to occur within these scaffolds. Overall, scaffolds are usually porous and created by various methods, such as electrospinning, phase-separation, freeze-drying, and self-assembly [3]. They enhance the body’s ability to heal itself by providing a biodegradable matrix that can enable cells to grow [1].

Electrospinning is a quick and efficient way to produce scaffolds because it allows the scientist to control parameters such as the sizes of nanofibers and nanopores [4]. Other parameters can be constructed as well based on a careful selection of the polymer and an appropriate solvent [5]. During electrospinning, the polymer will dissolve in a volatile solvent and be loaded into a syringe. This liquid is extruded from the needle tip at a constant rate by a syringe pump. In addition, a positive or negative lead will connect to the needle-tip of the syringe while a ground lead is placed on a collector plate [6]. The distance between the syringe-tip and the collector plate will vary depending on the properties of the polymer solution. When the electrostatic force on the polymer solution overcomes the surface tension, a jet of the polymer solution will form and eventually travel towards the collector plate [7]. As the jet flows towards the collector plate, the liquid will accelerate and deposit micro/nanofibers of the polymer on the collector plate [8]. 

Utilizing current methods, cells are seeded onto the scaffold after it has been formed. Cell seeding can be time-consuming because it requires three steps: creation of the scaffold, differentiation of the cells, and incorporation of the cells into the scaffold. Cell differentiation is already time-consuming and requires additional components, such as growth factors [9]. Another problem arises once these cells are differentiated and seeded: limited ability of cell diffusion into the scaffold [10]. Limited diffusion can produce a nonuniform distribution of cells that causes varied properties and cell densities within different areas of the scaffold. This is potentially detrimental to the longevity of the scaffold both in vitro and in vivo [11]. A possible method to increase cell dispersion in the scaffold is to directly incorporate the cells into the electrospinning process.

There is evidence suggesting that externally applied magnetic fields can affect cell differentiation. It is likely that the generated electric field affects the cell membrane [12]. When the membrane is forced to change shape, it will distort the cytoskeleton of the cell, which attaches the cell membrane to the nucleus. This change in the cytoskeleton will affect the expressed genes and cause the creation of different cell signals, which could induce differentiation [13]. Incorporating stem cells into the electrospinning process will expose them to an electric field that likely induces unique behaviors (i.e., cell differentiation) as previously reported while using lower voltages [14,15]. While this has the potential to be successful, there are many potential hazards to consider. 

A concern in this process is that cells could not survive the voltage used in electrospinning. While an electric field could cause unique behaviors, an excessively large electric field could be detrimental to the viability of the cells. Moreover, the field could denature specific protein channels in the membrane, which causes irreparable cell damage [16]. Voltages will be kept as low as possible to prevent this from occurring. Typical electrospinning voltages range from 1 kV to 30 kV [17]. The applied voltage required to create a scaffold will vary depending on the polymer used. 

Jayasinghe et al. made use of the coaxial electrospinning from immortalized human brain astrocytoma [18]; a year later, the author described its use from primary porcine vascular smooth muscle cells or rabbit aorta smooth muscle cells, while the protectant polymer was polydimethylsiloxane (PDMS) [19]. Yunmin et al. showed simultaneous bio-electro spraying of human adipose stem cells (ASCs) while electrospinning polyvinyl alcohol (PVA), but the study used two separate needles [20]. Recently, Hoare et al. used hydrazide-functionalized POEGMA (POH) and aldehyde-functionalized POEGMA (POA) along with poly(ethylene oxide) (PEO) to successfully encapsulate NIH 3T3 fibroblasts and electrospin them [21]. 

This research will test two different polymer combinations: collagen/poly(ethylene oxide) (PEO) and gelatin/pullulan. These polymer combinations will electrospin at around 8 kV of applied voltage. One other constraint for this experiment is the solvent used for dissolving the polymer. In current electrospinning methods, common solvents for collagen include 1,1,1,3,3,3-hexafluoro-2-propanol (HFP), 2,2,2-trifluoroethanol (TFE), or acids (tri-fluoro acetic acid (TFA), acetic acid, hydrochloric acid) [22,23]. These solvents could be toxic if cells are directly incorporated into the polymer–solvent solution. To overcome this restriction, the group will use cell media as the solvent. 

Collagen was chosen as the initial polymer because it is the primary constituent of the body’s natural extracellular matrix [24]. However, collagen is typically electrospun with an acetic acid solvent, which would likely cause cell death. No studies have attempted to show the success of electrospinning collagen with cell media as the solvent. Therefore, other polymers will also be utilized to determine which one creates the best scaffold. Moreover, collagen may degrade while electrospinning, but successful trials have been reported using different methods, including modifying the collagen surface with methyl methacrylate-*co*-ethyl acrylate [25]. Gelatin is simply denatured collagen; therefore, it can create scaffolds with the same success as collagen [26]. Because previous studies have determined that PEO increases the yield of uniform fibers when electrospun with other polymers, we decided to use it as well [27]. Pullulan and gelatin are commonly used together in hydrogels, and pullulan has shown antioxidant potential [28]. Therefore, the group will electrospin with pullulan, gelatin, or a combination of both. Adipose-derived stem cells (ADSCs) will be used due to their accessibility and potential for creating various terminally differentiated cells, such as osteoblasts, chondrocytes, adipocytes, and neurons [29]. The cells will be directly incorporated into the five polymer solutions prior to electrospinning. To group’s knowledge, this is the first time that electrospinning live cells using these natural biocompatible polymers has been reported.

## 2. Materials and Methods 

### 2.1. Electrospinning Device

Due to the nature of working with living stem cells, it was imperative to maintain sterile conditions throughout the entire spinning process. To maintain a sterile environment, the spinning took place under a sterile biological safety cabinet. The electrospinning device needed to withstand exposure to ultraviolet (UV) light, so it could be sterilized for at least 24 hours under the culture hood as the UV light was usually turned on the night before the experiment. Acrylic, which can handle UV exposure, was determined to be the material that the electrospinning device would be constructed with. Acrylic sheets and cement were used to construct the framework of the electrospinning device along with the necessary spinning supplies, such as a plate, voltage supply, electrical leads, and syringe pump. 

### 2.2. Cell Culturing and Electrospinning 

P_2_-P_4_ of adipose tissue-derived stem cells (hASCs) from Lonza (Walkersville, MD, USA) were used for cell cultures. Cells were plated in T75 culture-treated flasks with approximately 1 million cells per flask, and culture media was changed every 3-4 days for the duration of the culture. Three components make up the cell electrospinning solution: protectant, solvent, and cell pellet. Collagen, Poly(ethylene oxide 10,0000), pullulan, and gelatin powders were used as protectants. Poly(ethylene oxide) (Sigma), pullulan (Hayashibara Laboratories, Okayama, Japan), Type A gelatin from porcine skin (Electron Microscopy Sciences, Hatfield, PA, USA), and extracted collagen from rat tail were dissolved in solvent at concentrations of 2.5 mg/mL, 5 mg/mL, 10 mg/mL, 20 mg/mL, and 30 mg/mL. For PEO, gelatin, and pullulan, the powder was dissolved in serum-free culture media in the previously mentioned concentrations, while collagen acetic acid was used instead of serum-free culture media. The protectants and mesenchymal stem cell medium (solvent) were mixed again at the ratio of 1:1 by volume and placed on a stirring hot plate for 20–30 minutes to warm and mix. The solution was warmed to 40 degrees Celsius in the case of gelatin. The tube temperature reduced to 37 degrees Celsius. Cell pellets (1 × 10^6^) were then added to the protectant solution. Cell electrospinning content was aseptically transferred to a sterile 10 ml syringe, and a sterile 18-gauge syringe needle tip was secured. The collector plate, which is a petri dish, was positioned 7.5 cm from the end of the needle tip. The syringe pump settings were adjusted to produce readings for a plastic 10 ml syringe pump. The pump rate was set to 30 μL/min and reduced at increments of 5 μL/min to determine the optimized pump rate for each cell electrospinning solution. Control cells using the same combination of gelatin, pullulan, gelatin/pullulan, collagen, or PEO were sprayed at the same rate on an empty petri dish without any voltage application. This procedure was done at room temperature, and electrospinning was never performed for more than 15 minutes. 

### 2.3. Viability Test

The viability was investigated by a live/dead assay kit and fluorescence microscopy. Approximately 6 hours after electrospinning, the culture media was aspirated from each well. After incubation with calcein and ethidium (2 μM calcein and 4 μM ethidium in PBS) for 10 minutes at 37 °C, samples were washed with PBS and cells were imaged.

#### Cytotoxicity Test (Lactate Dehydrogenase (LDH) Activity)

The media was aspirated two days after spinning, and cells were washed with PBS. Lactate dehydrogenase or LDH (Cytotox96 kit, Promega, Madison) was performed on the attached cells according to the manufacture’s protocol to look at the cell viability using cell lysate [30].

$$\mathrm{Viability}\%=\frac{Average\;OD\;of\;sample\;*\;100}{Average\;OD\;of\;control}$$

### 2.4. Gene Expression by Reverse Transcription-Polymerase Chain Reaction (RT-PCR)

Seven days after spinning, RNA was isolated according to the manufacturer’s instructions for the RNeasy plus mini kit (Qiagen, Germantown, MD, USA), and RT-PCR was performed according to the instruction manual of the One-Step RT-PCR kit (Qiagen, Germantown, MD, USA). The selected pluripotential genes were SOX2 and OCT4.

### 2.5. Immunocytochemistry

Cellular morphology was visualized on Day 2 using fluorescence microscopy. Briefly, samples were fixed with 4% paraformaldehyde (PFA) in PBS (pH 7.4) for 15 min at room temperature (RT). After rinsing with PBS three times, the samples were placed in a permeabilization solution with 0.1% (v/v) Triton X-100 for 10 min and rinsed again with fresh PBS three times. The cells were incubated with Phalloidin 488 and DAPI (Life Technologies, Carlsbad, CA, USA) to visualize the f-actin and nuclei, respectively. 

### 2.6. Microscopy

To observe the structure of the scaffold, Fluorescein Isothiocyanate (FITC)-conjugated gelatin was used. Electrospun cells/scaffolds deposited on microscope glass slides were imaged using an Olympus BX51 microscope equipped with an Olympus DP73 camera and CellSens software.

### 2.7. CytoViva Microscopy 

To confirm that the cells were embedded within the scaffold, the cells were labeled using a green CMFDA cell tracker dye (Invitrogen, Oregon, Germantown, MD, USA) before electrospinning; they were labeled with DAPI afterward. Samples were imaged using CytoViva’s patented enhanced darkfield transmitted light condenser (NA 1.2–1.4) coupled with CytoViva’s proprietary Dual Mode Fluorescence (DMF) module. These components were configured on an Olympus BX51 upright microscope using an Olympus100X oil UPL Fluorite objective (NA 0.60–1.30) with adjustable iris objective optimized for darkfield imaging. The light source used was Prior Lumen 200 with a metal halide lamp and variable light attenuation. Optical images were captured using a DAGE-MTI XLMCT cooled CCD camera with a 7.4 µm pixel size.

### 2.8. Fourier-Transform Infrared Spectroscopy (FTIR)

Scaffold compositions were determined by loading the samples onto an attenuated total reflectance (ATR) attachment and using a Thermo Scientific Nicolet iS 50 FTIR (Thermo Fisher, Waltham, MA, USA). Data were plotted in MS Excel (Microsoft, Redmond, WA, USA).

## 3. Results

**Figure 1:**
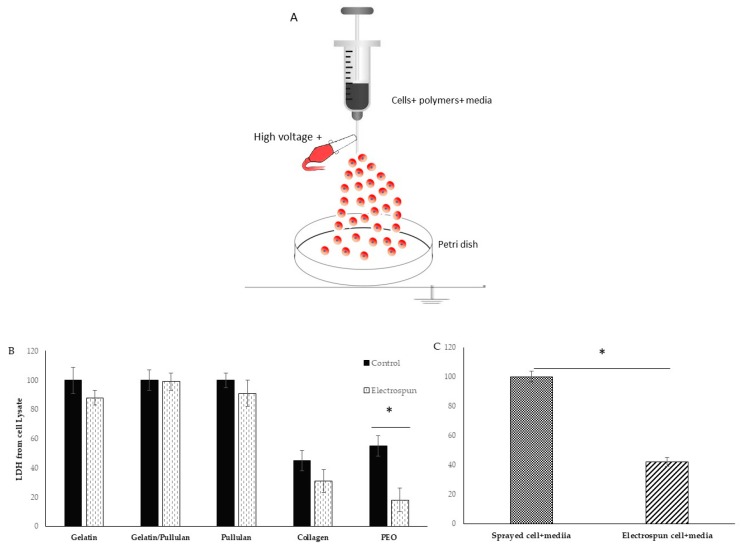
(**A**) Schematic diagram of electrospinning setup for live cells; (**B**) lactate dehydrogenase (LDH) from cell lysate in electrospun and control groups; (**C**) LDH from cell lysate in electrospun (cell+ media) compared to Sprayed cell+ media. Results are normalized to control. * shows *p*-value < 0.05.

A scheme of the electrospinning process is shown in Figure 1A. Electrospinning was only observed at a concentration of 5 mg/mL at 8 kV (Table 1). Cells were detected 6 hours after electrospinning to observe attachment as a sign of viability. Most cells in the collagen scaffold were dead (i.e., stained red). The PEO scaffold had a lot of red cells floating in the petri dish. Gelatin, pullulan, and pullulan/gelatin had good cell viability (i.e., stained fluorescent green), while the number of dead cells (stained red) was minor. 

The viability of cells in collagen was very low in both control and electrospun groups. Control cells were sprayed at the same rate on the petri dish without any voltage application. The 0.01% acetic acid that was used to dissolve collagen is probably the reason for low cell viability. PEO was dissolved in cell media and is biocompatible. However, very low cell attachment was observed in both control and electrospun groups, but the group realized this did not result from just the PEO. The PEO viability was 50% in the control group and less than 20% in the electrospun group. There is a significant difference between the control and electrospun group which is caused by the additional effect of electrospinning. In the electrospinning process, the polymer solution is exposed to shear stress, and dead cells in PEO can be the result of non-Newtonian fluids’ behaviors and shear stress. When PEO was removed from the formulation, gelatin/cells’ viability and attachment were good, and LDH on cell lysate showed 88% cell viability from the electrospun group compared to the control group (Figure 1B,C). However, when the sample was switched to pullulan/gelatin/cells, the group achieved 99% viability compared to the control, but the pullulan/cell scaffold had 91% viability. The *p*-value was not statistically significant among the three groups. To prove the role of the protectants (gelatin and pullulan), cells were electrospun with only culture media, and the cell viability reduced to 40% compared to cells and media that were sprayed with the same rate on the petri dish. 

**Figure 2:**
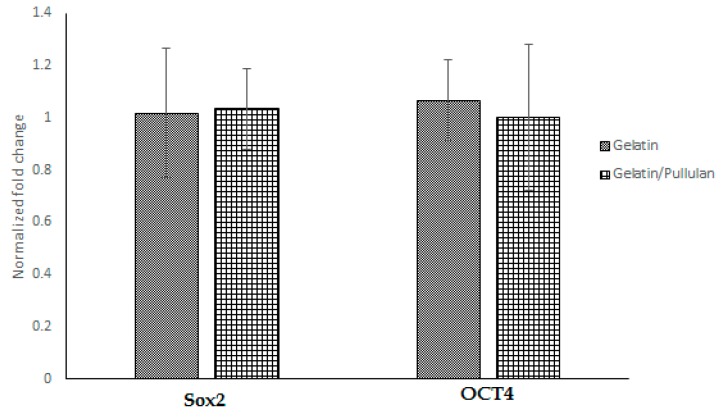
Gene expression in gelatin and gelatin/pullulan electrospun groups normalized to control. Control groups are cells cultured with 5 mg/mL gelatin or 5 mg/mL pullulan/gelatin.

Seven days after electrospinning, Oil Red O, toluidine blue, and Alizarin Red S staining were used to study adipogenic, chondrogenic, and osteogenic differentiations. All cells were negative for Oil Red O, toluidine blue, and Alizarin Red S. Moreover, PCR data showed no significant change in SOX2 and OCT4 after electrospinning (Figure 2), which confirms stemness after and before electrospinning. 

**Figure 3:**
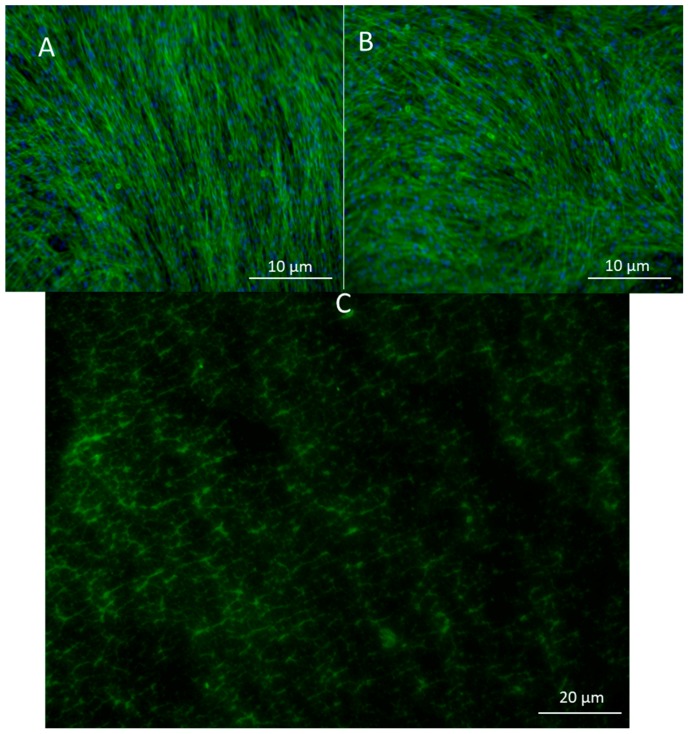
(**A**) Actin staining of adipose-derived stem cells in control and in (**B**) pullulan/gelatin/cells at 10×. Phalloidin 488 (green) labels actin, while DAPI (blue) labels the nucleus. (**C**) Cells were surrounded by FITC (green) conjugated gelatin.

To look at the cell alignment, the group used actin staining 2 days after electrospinning. Cell alignment was random as expected (Figure 3A,B). Images of the scaffold with FITC gelatin showed a porous structure which was later confirmed by CytoViva imaging as well. (Figure 3C). 

A highly porous structure was observed after CytoViva imaging. It appears that the cells become embedded in these pores as confirmed by another CytoViva imaging, where cells were pre-stained with a cell tracker and DAPI. Those that house the cells were approximately 10 µm in diameter. 

The band observed at 996 cm^−1^ in the pullulan and electrospun samples, which is associated with C-OH bending vibrations at the C-6-position in the case of polysaccharide, indicates the strength of the interchain interactions via hydrogen bonding [31]. 

**Figure 4:**
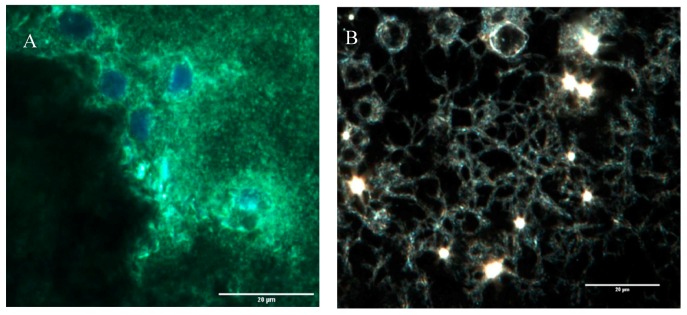

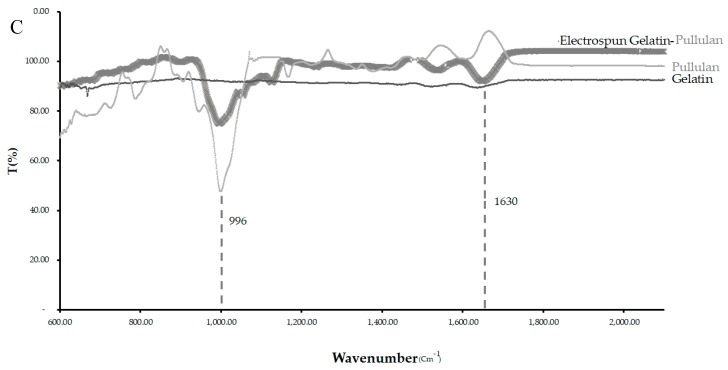
CytoViva and FTIR of the three scaffolds. (**A**) The cells were stained with CellTracker Green CMFDA (Invitrogen) at 2.5 μM for 1 h before electrospinning and were stained for DAPI after electrospinning. (**B**) CytoViva image of the cells and scaffold with no pre-staining. (**C**) FTIR of the pullulan, gelatin and gelatin/pullulan electrospun scaffolds.

The primary hydroxyl groups at the C-6-position were available in the pullulan macromolecule (Figure 4C). However, there were no hydroxyl groups at the C-6-position in gelatin. This band can show the glycosylation between the gelatin and pullulan molecules or the formation of the interchain hydrogen bond in the composite fiber. The amide I (AmI) band at 1630 cm^−1^ in pullulan/gelatin was the strongest among the three and shifted slightly to a higher wavelength, which can be associated with AmI sensitivity to hydrogen bonding at the C=O group [32,33]. Hydrogen bonding plays a significant role in the stabilization of protein secondary structure which can result from the presence of pullulan here [34].

This experiment was run at 8 kV, and the best concentration for Pullulan/gelatin was 5 mg/ml at the ratio of 1:1. Further studies are needed to look at the effect of higher voltage on cell viability and differentiation. 

## 4. Discussion

Uniaxial electrospinning with a single needle is a common technology for the fabrication of scaffolds that can provide the initial scaffold for tissue engineering applications. On the other hand, coaxial electrospinning facilitates the incorporation and preservation of bioactive substances, where the shell is often used to protect sensitive substances encapsulated in the core. In this new method, uniaxial electrospinning is incorporated with live cells. Polymers are used to protect the cells, and the cells are encapsulated in the polymers during the electrospinning process. In this study, the group used pullulan, gelatin, collagen, and PEO. Trials with collagen and PEO were unsuccessful as cell viability was not acceptable. Highly hydrated polymers, such as PEO, suppress cellular and molecular adhesions by providing a physical steric barrier [35,36].

This study proved that pullulan and gelatin could protect cells from high voltage damages. Pullulan and gelatin are biocompatible, water-soluble polymers that have been shown to be ineffective at changing phenotype, viability, and cell differentiation. Pullulan can quench reactive oxygen species [37] and be a great scaffold in combination with gelatin. Moreover, pullulan can increase the tensile strength of gelatin, which is very important in tissue engineering [38]. Its structural features, such as the presence of large amounts of hydroxyl groups in the main chain, make it an optimal polymer for creating scaffolds. Studies show that the increase in the pullulan content of a scaffold leads to an increase in viscosity and eventually a decrease in electrical conductivity [39]. The group believes that the composition used in this experiment for making scaffolds acted as a shield for live cells against electrical conductivity, which was shown by the viability studies.

CytoViva images showed a porous structure with cells embedded in it. Moreover, we observed single cells covered by pullulan and gelatin, which was confirmed by fluorescence microscopy. Studies have shown that integrin is an electric field-sensing protein on the cell surface [40]. In addition, gelatin attaches to cells via integrin. Blocking the sensing proteins may be the reason for protecting the cells from high-voltage damage. In general, gelatin can protect the cells by covering the essential structures required for cell function and viability [41]. Gelatin and pullulan are both water-soluble, and this scaffold will dissolve after incubation at 37 °C. These polymers protect the cells during electrospinning but eventually dissolve in the media which can be altered by biocompatible crosslinkers [42].

In this study, no significant difference was found in Sox2 and Oct4 gene expression before and after electrospinning, but it has been shown by numerous studies that low voltage electrical stimulation can affect gene expression of transforming growth factor-β (TGF-β), collagen type-I, alkaline phosphatase (ALP), bone morphogenetic proteins (BMPs), and chondrocyte matrix [43]. More experiments are needed to better understand the effect of 8 kV on stem cells, and the broader gene expression still needs to be studied. 

## 5. Conclusions

The success of this project opens a new field of study within tissue engineering. The discovery that cells can be directly incorporated into the electrospinning process has many potential benefits within the tissue engineering realm. In this paper, electrospinning with an applied voltage of 8 kV was observed at a concentration of 5 mg/mL gelatin/pullulan, but there are many combinations of polymers and cell-types that can still be tested. The application of this design is endless, and many properties of cells may change with variations of voltages and materials. Although this method is limited to water-soluble polymers, using the core–shell technology allows the use of other polymers. By using the core–shell technology, the outer polymer can be loaded with cells and the inner core can be loaded with a polymer that is not water-soluble.

## Figures and Tables

**Table 1:** Different concentrations that have been tried to electrospin cells.

Polymer	Concentration	Viability from Dead/Live
PEO	2.5 mg/mL, 5 mg/mL, 10 mg/mL, 20 mg/mL, and 30 mg/mL	Not acceptable
Collagen	2.5 mg/mL, 5 mg/mL, 10 mg/mL, 20 mg/mL, and 30 mg/mL	Not acceptable
Gelatin	2.5 mg/mL, 5 mg/mL, 10 mg/mL, 20 mg/mL, and 30 mg/mL	Acceptable
Pullulan	2.5 mg/mL, 5 mg/mL, 10 mg/mL, 20 mg/mL, and 30 mg/mL	Acceptable
Gelatin/ Pullulan	2.5 mg/mL, 5 mg/mL, 10 mg/mL, 20 mg/mL, and 30 mg/mL	Acceptable
